# A Case of Spinal Epidural Abscess and Paraplegia After Group A Streptococcal Pharyngitis

**DOI:** 10.3390/idr18040068

**Published:** 2026-07-04

**Authors:** Blake J. McKinley, Rob M. Seby, Robert C. Chase, Tatjana Gavrancic, Jeremy Collado, Libardo Rueda Prada

**Affiliations:** 1 Department of Internal Medicine, Mayo Clinic, Jacksonville, FL 32224, USA; 2 Department of Hospital Internal Medicine, Mayo Clinic, Jacksonville, FL 32224, USA; 3 Department of Emergency Medicine, Mayo Clinic, Jacksonville, FL 32224, USA

**Keywords:** streptococcal infections, *Streptococcus pyogenes*, pharyngitis, epidural abscess

## Abstract

**Background**: Spinal epidural abscesses (SEA) are rare, occurring 2.5–3 times per 10,000 hospital admissions. While streptococcus species comprise 7% of reported SEAs, Group A Streptococcus (GAS) has been described only once in the medical literature to our knowledge. **Case presentation**: We present a case of GAS pharyngitis with subsequent paraplegia from a GAS SEA. A 33-year-old female presented to the emergency department (ED) and was initially diagnosed with GAS pharyngitis and a suspected strained lower back. Following treatment with amoxicillin, she returned with worsened back pain, radiculopathy, and leukocytosis, for which she was treated with cyclobenzaprine. On her third presentation, she had new bilateral lower extremity weakness with decreased sensation, bilateral ankle clonus, and hyperreflexia. MRI of the thoracic and lumbar spine revealed a multiloculated SEA at T5-T10 requiring laminectomy and abscess evacuation. Intraoperative cultures grew *Streptococcus pyogenes*. Despite surgery, medical management, and physical therapy, she remained paraplegic. **Conclusions**: To our knowledge, this is the first report of SEA preceded by GAS pharyngitis. This case exposes the critical association between a recent infection, progression of back pain, eventual neurologic symptoms, and inflammatory markers that should trigger concern for SEA and early evaluation with MRI.

## 1. Introduction

Spinal epidural abscess (SEA) is a rare infectious process that results in a purulent collection between the dura mater and the spinal column, which can compress the spinal cord and lead to devastating and sometimes permanent neurological deficits or even death. The lower thoracic and lumbar regions are affected most commonly, causing paraplegia; however, cases of cervical spine involvement can occur, leading to quadriplegia [1]. When paraplegia or quadriplegia has occurred, it is a surgical emergency [2]. SEA incidence is more common in adults and is reported as occurring 2.5–3 per 10,000 hospital admissions [3]. *Staphylococcus aureus* is responsible for most cases of SEA, accounting for an estimated two-thirds of cases [4], followed by coagulase-negative *Staphylococcus*, and Gram-negative bacilli [5]. *Streptococcus* species are reported to cause 7% of SEA [6], including *intermedius* [7,8], *gallolyticus* [2], *constellatus* [1,9], *viridans* [10], and *pneumoniae* [11]. A spontaneous case of SEA was reported in a 22-month-old female from Group A Streptococcus (GAS), also known as *Streptococcus pyogenes* [12]. Here we present a rare case of an immunocompetent female who experienced GAS pharyngitis and subsequently developed a SEA and paraplegia. Unlike the previously reported pediatric case, our patient was an immunocompetent adult who developed severe neurologic complications, highlighting the rarity of GAS-associated SEA in adults.

## 2. Case Presentation

A 33-year-old female presented to the emergency room (ER) with a chief complaint of severe worsening mid-lower back pain, bilateral lower extremity numbness, tingling, and weakness that limited her ambulation, associated with urinary hesitancy and a sensation of bladder fullness for less than 1 day. She denied fecal or urinary incontinence, skin rash, fever, or chills. Her past medical history was remarkable for hypothyroidism, bipolar disorder, and opioid use disorder on buprenorphine-naloxone. She denied drug, alcohol, or tobacco use. The patient had presented to the ER twice in the last 3 weeks (see Table 1). First, she presented with new-onset sore throat and five days of mild back pain 17 days prior. She was diagnosed with possible mechanical back strain and group A streptococcal (GAS) pharyngitis by a positive rapid strep test. She was treated with 10 days of amoxicillin and cyclobenzaprine as needed. In the ER presentation, 14 days later, she was evaluated for worsening back pain without radiculopathy. She had no neurological deficits on physical exam. At that time, her laboratory and imaging workup showed leukocytosis of 18.6 × 10^9^/L, elevated D-dimer of 534 ng/mL, and a CT chest angiogram negative for pulmonary embolism or other acute process. Her back pain was considered musculoskeletal in nature, and she was discharged with a new prescription of cyclobenzaprine as needed.

On the patient’s third presentation, she had a temperature of 36.3 °C, pulse rate of 104 bpm, and blood pressure of 152/95 mmHg. Her physical examination was notable for bilateral lower extremity weakness (left lower extremity: 1/5 hip flexion, 2/5 knee flexion, 2/5 knee extension, 2/5 foot dorsiflexion, 4/5 foot plantiflexion; right lower extremity: hip flexion 2/5, knee flexion 3/5, knee extension 4/5, foot dorsiflexion 2/5, foot plantiflexion 4/5), sensory loss (decreased to fine touch bilaterally, increased sensation to pinprick in the left lower extremity, no vibration sensation bilaterally, decreased temperature sensation in the right lower extremity), and hyperreflexia 3/4 with sustained bilateral ankle clonus. There was no tenderness to palpation of the spine.

Her initial laboratory workup was remarkable for leukocytosis of 14.4 × 10^9^/L with neutrophilia, ESR 103 mm/h, C-reactive protein 155.3 mg/L, and urine drug screen positive for buprenorphine (chronic medication), hydromorphone (received during admission), and tetrahydrocannabinol (THC). A bladder ultrasound showed a distended bladder with an approximate volume of 1 L. An emergent CT of the thoracic and lumbar spine with IV contrast did not reveal any significant acute abnormality. However, MRI of the thoracic and lumbar spine with and without IV contrast revealed a large posterior epidural fluid collection suggestive of a multiloculated abscess extending from T5 to T10 with marked cord compression (Figure 1). Blood cultures were collected before initiation of intravenous vancomycin and ceftriaxone.

Neurosurgery performed emergent T5-T10 laminectomies, revealing brisk extrusion of copious purulent material at each level. Intraoperative cultures grew GAS with speciation of *pyogenes* on four separate swabs. The *Streptococcus pyogenes* cultures were found to be susceptible to ceftriaxone. Throughout the patient’s hospital stay, no bacteremia was identified on a total of six blood culture bottles (three sets, two bottles per set). One set was collected on admission (1 day before the procedure), and two postoperative sets were obtained on day 7 for worsening leukocytosis and on day 16 following a transient episode of fever and chills; all blood cultures remained negative. Additional physical examination and imaging showed what appeared to be several dental caries, but CT of the maxillofacial sinuses was negative for sinusitis or abscess. The patient had negative screening tests for *M. tuberculosis*, syphilis, and HIV, and her immunoglobulin levels were within normal ranges.

The patient remained hospitalized for 20 days and completed a dexamethasone taper over 14 days, starting at 6 mg every 6 h. The patient was continued on intravenous ceftriaxone 2 g every 24 h with plans to continue for 6 weeks. At discharge, the patient remained paraplegic from T8 down, with no mobility or feeling sensation in her lower extremities, and required a chronic urinary Foley catheter. Patient was discharged to a longer-term care facility for rehabilitation. Unfortunately, at the time of this report, the patient remained paraplegic from T8 down.

## 3. Discussion

SEA may occur from hematogenous spread, iatrogenic inoculation, or direct extension [13]. Hematogenous spread may arise from primary infectious sources such as skin and soft tissue infections (most commonly, including pharyngeal abscesses), dental abscesses, the urinary tract, endocarditis, previous sepsis, or IV drug use [3]. IV drug use has been reported to be the most frequently associated risk factor [14]. The underlying bacterial source cannot be identified in 30–40% of cases and is referred to as silent bacteremia [12]. Additionally, prior antibiotic exposure may reduce the diagnostic yield of blood cultures by suppressing bacterial growth, resulting in persistently negative cultures despite ongoing infection. Iatrogenic inoculation may occur from spinal surgeries, paraspinal injections, and placement of epidural catheters or stimulators [15]. Direct extension may occur in vertebral osteomyelitis, psoas abscess [3], or spinal abscess [15]. Patients with diabetes, HIV, or immunocompromised hosts are at a higher risk [5,16].

Our patient had no significant medical history, chronic infections, or recent procedures that would have predisposed her to a SEA. The patient’s opioid abuse history put her at risk for potential IV drug use. However, at the beginning of the admission, her urine drug testing was only positive for buprenorphine (chronic medication), hydromorphone (received during admission), and THC. The patient followed up with pain medicine specialists to manage her buprenorphine-naloxone use with regular drug testing. It is unlikely that IV drug use played a role in this case. Alcohol is also a risk factor for SEA [3]; however, the patient had no recent alcohol use. She did have dental caries, but no evidence of abscess formation on CT imaging. Our patient had a negative screening for HIV and no immunoglobulin deficiency to suggest an underlying immunodeficiency. The most significant history was the patient’s recent GAS pharyngitis. The surgical cultures confirmed a *Streptococcus pyogenes* infection, making the patient’s upper respiratory illness the most likely source of epidural seeding. GAS pharyngitis is usually considered a localized infection; however, *Streptococcus pyogenes* is known to cause invasive diseases, such as pneumonia, bacteremia, necrotizing fasciitis, and toxic shock syndrome [17]. To our knowledge, this is the first report of GAS pharyngitis being complicated by SEA. We assume that bacterial seeding took place via hematologic spread, although repeat blood cultures remained negative on admission and throughout the patient’s hospital stay.

The patient’s initial presentation included back pain at the time of the GAS pharyngitis diagnosis (17 days prior to SEA diagnosis). At that time, the patient described 5 days of symptoms prior to starting the 10 days of amoxicillin. Hematological spread likely occurred early in the disease course. There is some data to suggest that amoxicillin may treat GAS bacteremia [17], although the dosage required is much higher than standard doses for GAS pharyngitis treatment. 

When clinically diagnosing SEA, the literature commonly refers to the classic triad of sepsis/fever, back pain, and neurologic deficit [3,5,12]. However, these symptoms have been reported as occurring together only 8% of the time, with fever present in less than half of patients and neurological deficits have been reported in 33–50% of patients [5,18]. The patient’s initial symptom of back pain is the most common symptom, occurring in 75% of patients with SEA. The patient lacked spinal tenderness (present in 58% of patients), but eventually experienced motor weakness, paralysis, and bladder dysfunction, occurring in 40%, 27%, and 27% of patients, respectively [3]. This case highlights the importance of screening for bladder dysfunction with a bladder volume measurement. Radicular pain and nuchal rigidity are often described prior to motor weakness [14]. It is unclear if the patient experienced any of these symptoms prior to presenting to the ED. The initial presentation never included fever.

The most important prognostic factor for a favorable outcome is early diagnosis [3]. Non-specific presentations commonly lead to a delay in the diagnosis of SEA, with misdiagnosis occurring in as many as 90% of patients in their first ER visits [18]. Another study showed that 75% of patients had a delay in diagnosis of SEA, defined as requiring multiple ED visits before diagnosis or being admitted for greater than 24 h with no definitive diagnostic study [19]. Gadolinium-enhanced MRI is the most sensitive and specific diagnostic study but is time-intensive, costly, and not the study of choice for evaluation of acute low back pain lacking alarm features [3]. Furthermore, emergency medicine physicians are encouraged to be selective with their advanced imaging for lower back pain, as it is among the 4 most common reasons to present to the ED for adults aged 18–65 years [20]. Guidelines from the American College of Radiology and the American College of Emergency Physicians advise against imaging in general, except for cases in which there are red flags, such as neurological compromise or concern for fracture, malignancy, or infection [20]. The national Choosing Wisely campaign is a U.S. initiative to reduce unnecessary costs and radiation for patients by reducing low back pain imaging in the ED [21]. ED physicians face a diagnostic dilemma when deciding which patient requires imaging for non-specific presentations of lower back pain, as described in the early presentation of this case. 

Our patient initially presented 17 days prior to admission with back pain and no fever or neurological deficit. The only laboratory evaluation performed was a positive rapid strep test. The follow-up ED visit 3 days prior to admission was significant for unexplained leukocytosis and did involve advanced imaging (although not dedicated to the spine) with a CT chest angiogram that did not reveal acute abnormalities. The day of hospital admission, the patient had leukocytosis and elevation of inflammatory markers compatible with a significant ongoing inflammatory process. The follow-up imaging with dedicated CT scans of the thoracic and lumbar spine did not identify the epidural abscess, but it was identified with MR imaging. This case helps highlight the importance of investigating non-specific back pain with inflammatory laboratory metrics and follow-up with gadolinium-enhanced MRI if values are elevated and SEA is on the differential. ESR has been shown to have 100% sensitivity and 67% specificity for SEA [5]. A prospective study investigated the implementation of a novel decision guideline using risk factor assessment, followed by ESR and CRP testing, prior to definitive imaging. Before guideline implementation, diagnostic delays were observed in 46 (83.6%) of 55 patients versus 3 (9.7%) of 31 after guideline implementation (*p* < 0.001) [22]. This shows that education on pertinent signs and symptoms of patients with SEA and inflammatory marker evaluation can better characterize patients at risk for SEA.

Upon diagnosis of a SEA, blood cultures should be drawn immediately, and antibiotics should be started without delay and tailored to the offending organism’s antibiotic sensitivity. An antibiotic duration of 4 to 8 weeks is recommended to address the concurrent risk of osteomyelitis [23]. The use of corticosteroids, usually dexamethasone, has been used to reduce inflammation in patients with SEA, especially patients that are more extensively affected, although insufficient data are available to recommend their use [24]. 60% of patients are treated surgically, with a significant increase in surgical intervention in those with neurological deficits [6]. Irreversible paralysis affects 4–20% of patients [14]. The prognosis for our patient to recover movement or sensation in her lower extremities remains guarded, as she displayed no clinical improvement over 20 days despite aggressive physical therapy while hospitalized and completion of her antibiotic treatment course.

## 4. Conclusions

SEA may occur from GAS (*Streptococcus pyogenes*) pharyngitis. Diagnostic delays may lead to poor outcomes, including paraplegia. Clinicians should include SEA in the differential diagnosis of patients who present with back pain in the setting of a recent infection, and obtain a detailed history and physical to screen for signs and symptoms that may increase suspicion for SEA. Inflammatory markers such as WBC count, CRP, and ESR should be included in any diagnostic evaluation when SEA is being considered. A gadolinium-enhanced MRI is the most sensitive and specific diagnostic study for SEA, whereas CT imaging may not identify SEA. Blood cultures, antibiotics, and neurosurgical evaluation should not be delayed when SEA is identified.

## Figures and Tables

**Figure 1:**
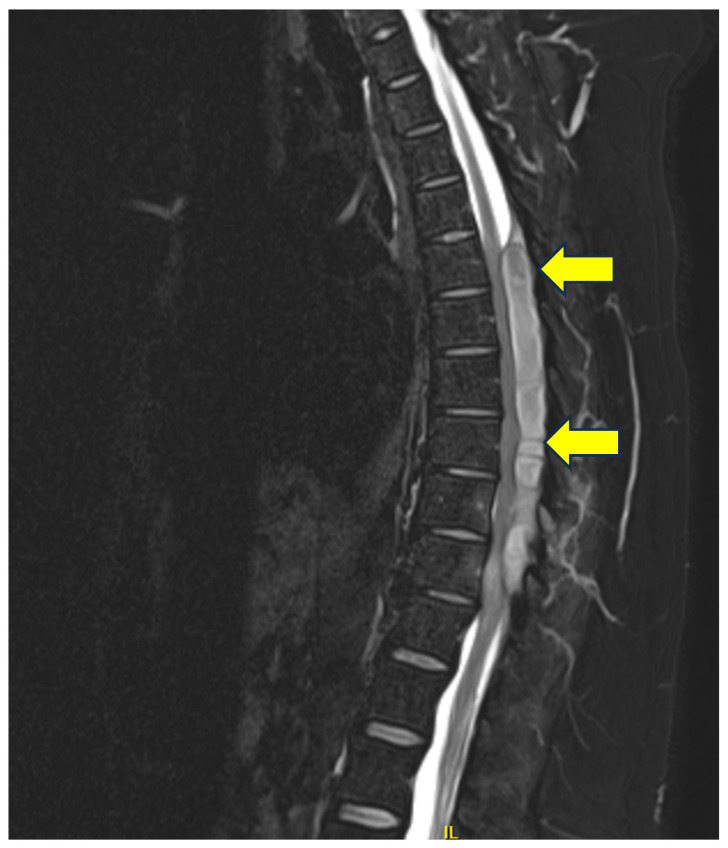
Sagittal T2-Weighted DIXON turbo spin-echo-sequenced MRI of the thoracic and lumbar spine revealing a multiloculated spinal epidural abscess (yellow arrows) extending from T5–T10 with marked compression of the thoracic cord.

**Table 1:** Clinical timeline of case presentation.

Timeline	Clinical Events
17 days before admission	ER visit for sore throat and mild back pain. Diagnosed with GAS pharyngitis (positive rapid strep test). Treated with amoxicillin.
3 days before admission	Returned with worsening back pain. Neurological examination was normal. CT chest angiogram was negative. Discharged with presumed musculoskeletal pain.
Day 0 (admission)	Presented with rapidly progressive bilateral lower extremity weakness, sensory loss, urinary retention, and inability to ambulate. MRI showed a T5–T10 spinal epidural abscess with cord compression.
Day 0	Emergent T5–T10 laminectomy with drainage. Intraoperative cultures grew Group A Streptococcus (Streptococcus pyogenes); blood cultures remained negative.
Hospital course/Discharge	Treated with ceftriaxone (planned 6 weeks) and dexamethasone taper. Discharged after 20 days to rehabilitation with persistent paraplegia and neurogenic bladder

## Data Availability

The data generated and analyzed during this study are not publicly available due to patient privacy and ethical restrictions. De-identified data may be available from the corresponding author on reasonable request, subject to institutional review and approval.
